# Annotating Cancer Variants and Anti-Cancer Therapeutics in Reactome

**DOI:** 10.3390/cancers4041180

**Published:** 2012-11-08

**Authors:** Marija Milacic, Robin Haw, Karen Rothfels, Guanming Wu, David Croft, Henning Hermjakob, Peter D’Eustachio, Lincoln Stein

**Affiliations:** 1 Informatics and Bio-computing Platform, Ontario Institute for Cancer Research, Toronto, ON, M5G0A3, Canada; E-Mails: MOrlic-Milacic@oicr.on.ca (M.M.); Karen.Rothfels@oicr.on.ca (K.R.); guanmingwu@gmail.com (G.W.); lincoln.stein@gmail.com (L.S.); 2 European Bioinformatics Institute, Wellcome Trust Genome Campus, Hinxton, Cambridge, CB10 1SD, UK; E-Mails: croft@ebi.ac.uk (D.C.); hhe@ebi.ac.uk (H.H.); 3 Department of Biochemistry, NYU School of Medicine, New York, NY 10016, USA; E-Mail: Peter.D’Eustachio@nyumc.org

**Keywords:** pathway database, pathway visualization, network visualization, cancer annotation, EGFR signaling

## Abstract

Reactome describes biological pathways as chemical reactions that closely mirror the actual physical interactions that occur in the cell. Recent extensions of our data model accommodate the annotation of cancer and other disease processes. First, we have extended our class of protein modifications to accommodate annotation of changes in amino acid sequence and the formation of fusion proteins to describe the proteins involved in disease processes. Second, we have added a disease attribute to reaction, pathway, and physical entity classes that uses disease ontology terms. To support the graphical representation of “cancer” pathways, we have adapted our Pathway Browser to display disease variants and events in a way that allows comparison with the wild type pathway, and shows connections between perturbations in cancer and other biological pathways. The curation of pathways associated with cancer, coupled with our efforts to create other disease-specific pathways, will interoperate with our existing pathway and network analysis tools. Using the Epidermal Growth Factor Receptor (EGFR) signaling pathway as an example, we show how Reactome annotates and presents the altered biological behavior of EGFR variants due to their altered kinase and ligand-binding properties, and the mode of action and specificity of anti-cancer therapeutics.

## 1. Introduction

The development of a malignantly transformed cell from a normal cell is a complex multi-step process that remains incompletely understood [1,2]. “Bottom-up” studies of relevant processes such as control of cell division, cell migration, tissue remodeling, and cell death have allowed the identification and characterization of many individual genes whose malfunction due to mutation or misregulation is associated with malignant transformation [3,4,5,6]. More recently, the development of high-throughput studies that exploit the availability of whole-genome sequencing has enabled “top-down” studies to systematically catalogue somatically mutated genes and altered patterns of gene expression in individual tumors [7,8]. These studies have confirmed the importance of genes identified as key players in the “bottom-up” studies, but have also suggested roles for additional genes and gene combinations not previously associated with processes relevant to malignancy.

Pathway databases have been effectively used to annotate our “bottom-up” understanding of molecular details of processes relevant to cell growth, differentiation, migration, and death. Here, we describe one such database, Reactome, focusing on extensions to this basic annotation strategy to allow the capture of details of disease processes, and on the development of data analysis tools to support the annotation and interpretation of gene sets identified in top-down studies.

Reactome is an open-source, open access, curated and peer-reviewed biological knowledgebase of human reactions, pathways and processes that serves as a platform for pathway visualization and analysis [9,10,11,12]. Reactome provides information about proteins and small molecules and how they participate in pathways to coordinate cellular events. The Reactome database employs a reductionist data model, which represents biology as reactions that convert input physical entities into output physical entities. The Reactome definition of a “reaction” is broad, including binding, dissociation, translocation and degradation, in addition to biochemical transformations of proteins and small molecules. Reactions are linked in causal chains to form pathways which in turn are grouped to represent larger biological processes like intermediary metabolism, innate immunity, solute transport, GPCR signal transduction, and apoptosis [13,14].

Reactome curators, in collaboration with outside expert researchers, annotate new pathways. The molecular details of every reaction are traceable to experimental evidence in the primary literature. If an event has not been directly studied in human systems, the appropriate non-human reaction is annotated and the homologous human one is inferred from it. Every pathway module is peer-reviewed by an additional expert. New and revised modules are publicly released to the Reactome website every quarter. Pathways, reactions, protein and small molecule entities are cross referenced with accession numbers and identifiers to a number of well-established databases, including NCBI Gene [15], Ensembl [16] and UniProt databases [17], UCSC Genome Browser [18], and ChEBI [19]. Physical entities and events are further linked to “Molecular Function”, “Biological Process” and “Cellular Component” ontology terms found in Gene Ontology (GO) [20].

Currently, the pathways in Reactome cover about 25% of the gene products encoded in the human genome, and contain the normal versions of many pathways that can be abnormally activated in cancer, such as “Signaling by EGFR” [21], “Signaling by FGFR” [22], “Signaling by NOTCH” [23], “PIP3 Activates AKT Signaling” [24], “RAF/MAP Kinase Cascade” [25]. We have also annotated a number of pathways that can be inactivated in cancer, such as pathways involving TP53: “Apoptosis” [26] and “Cell Cycle Checkpoints” [27], as well as pathways involving the RB1 protein family: “Mitotic G1-G1/S phases” [28].

Here, we use the epidermal growth factor receptor (EGFR), fibroblast growth factor receptor (FGFR) and PI3K/AKT signaling pathways to illustrate Reactome annotation of cancer pathways. EGFR and FGFR are transmembrane receptor tyrosine kinases. EGFR is activated by several growth factors, including the epidermal growth factor (EGF) [29]. FGFR family members (FGFR1, FGFR2, FGFR3 and FGFR4) are activated by 18 of 22 existing human fibroblast growth factors (FGFs), with each FGFR showing different affinity for individual FGFs [30]. Growth factor binding induces a conformational change that enables dimerization and trans-autophosphorylation on C-tail tyrosine residues of EGFR [31] and FGFRs [32,33,34]. Phosphorylated tyrosines in the C-tails of EGFR and FGFR serve as docking sites for downstream effectors that, upon binding to phosphorylated receptors, activate intracellular signaling cascades that regulate cellular proliferation, differentiation, and survival [30,35,36]. One of the intracellular signaling cascades downstream of EGFR and FGFRs is PI3K/AKT signaling [37,38]. PI3K class IA enzymes are heterodimers composed of a regulatory subunit p85 (encoded by PIK3R1, PIK3R2 or PIK3R3) and a catalytic subunit p110 (encoded by PIK3CA, PIK3CB or PIK3CD) [39]. The catalytic p110 subunit of PI3K becomes activated when inhibitory contacts with the p85 regulatory subunit are relieved by p85 binding to phosphorylated adaptor proteins recruited to activated EGFR or FGFRs [40,41]. Active PI3K class I enzymes phosphorylate PIP2 (phosphatidylinositol-4,5-bisphophate), converting it into PIP3 (phosphatidylinositol-3,4,5-trisphosphate), a reaction negatively regulated by PTEN phosphatase [42]. PIP3 serves as a second messenger that activates AKT (AKT1, AKT2 or AKT3) [43]. AKT family members are cytosolic and nuclear serine/threonine protein kinases involved in phosphorylation-mediated regulation of numerous proteins involved in cell survival and growth [39].

EGFR, FGFRs, PIK3CA, PIK3R1 and AKT1 are proto-oncogenes, frequently activated in cancer through gain-of-function mutations and/or overexpression. PTEN is an established tumor suppressor gene, with a frequent loss of function in cancer [44]. Gain-of-function mutations in EGFR [45,46] and FGFRs [47,48,49,50,51] usually act by conferring ligand-independent activation or by increasing tyrosine kinase catalytic activity. Mutations in PIK3R1 or PIK3CA abolish inhibitory interactions between the regulatory and catalytic subunit of PI3K [52,53,54,55,56], resulting in PI3K activity in the absence of growth factor stimulation. AKT1 gain of function mutations increase AKT1 affinity for PIP2, allowing AKT1 activation in the absence of PI3K activity and PIP3 generation [57]. PTEN loss-of-function mutations usually affect the phosphatase domain, impairing PTEN catalytic activity and removal of PIP3 [58].

Small molecule therapeutics and recombinant antibodies are being developed as potential treatments for cancers driven by increased activity of EGFR, FGFR and/or PI3K/AKT. Gefitinib and erlotinib, small tyrosine kinase inhibitors, are approved for the clinical treatment of cancers harboring specific EGFR mutations. A recombinant antibody, cetuximab, is approved for the clinical treatment of cancers that overexpress wild-type EGFR [59]. Small molecules that inhibit the catalytic activity of FGFRs [60], PI3K and AKT [61] are currently undergoing clinical trials or are in pre-clinical development.

We have extended the Reactome data model and enhanced the web tools to permit the annotation and visualization of the altered biological behavior of protein variants. These enhancements can be applied to any molecular abnormality due to germline or somatic mutation, as well as to abnormalities due to expression of foreign proteins encoded by genomes of infectious agents like viruses or intracellular parasites.

## 2. Results and Discussion

### 2.1. Annotation of Cancer-Perturbed Pathways

Pathways that stimulate cell growth, cell division and survival, and maintenance of undifferentiated state are activated in cancer through gain-of-function mutations in participating proto-oncogenes and/or their overexpression. On the other hand, pathways that negatively regulate cell division, growth and survival, or promote cellular differentiation are inactivated through loss-of-function mutations in tumor suppressor genes and/or their downregulation. To capture these two groups of cancer effectors, we have added new classes of data to the Reactome database.

#### 2.1.1. Extension of Protein Modifications to Accommodate Annotation of Changes in Amino Acid Sequence

**Figure 1:**
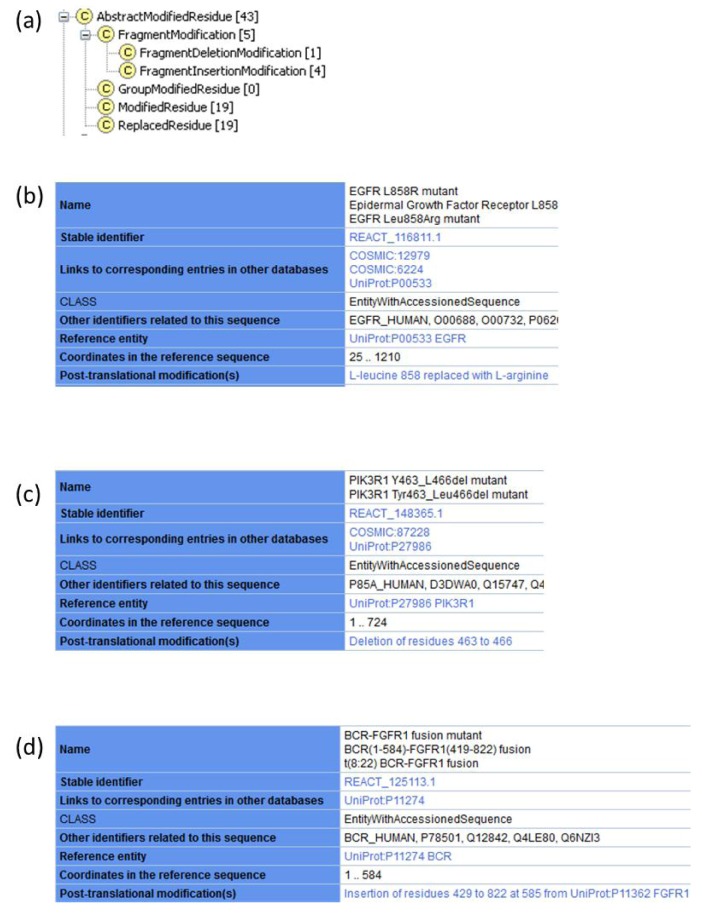
Annotation of cancer mutations. Reactome records are not displayed in their entirety due to space limitations. (**a**) Subclasses of protein modifications contained in Reactome class Abstract Modified Residue. Currently, Reactome website displays all subclasses of protein modifications in the single field “Post-translational modification(s)”. Future changes to the website will allow chemical modifications to be distinguished from effects of mutations. (**b**) Reactome record for EGFR L858R caused by a missense mutation that replaces leucine residue at position 858 with arginine. (**c**) Reactome record for PIK3R1 Y463_L466del caused by an in-frame intragenic deletion in PIK3R1 that removes amino acid residues from position 463 to position 466, as captured by the Fragment Deletion Modification instance. (**d**) Reactome record for BCR-FGFR1 fusion protein. Truncation of the wild-type BCR protein sequence is shown by altered end coordinate. FGFR1 fragment fused to BCR is annotated as an insertion using FragmentInsertionModification class.

The protein modification class in the Reactome data model was constructed to support annotation of covalent co- and post-translational modifications of proteins such as the phosphorylation of serine residues. To allow for annotation of mutant proteins, two new subclasses of modifications were introduced: Replaced Residue and Fragment Modification (Figure 1a). The Replaced Residue class is used to annotate amino acid substitutions in a protein sequence. A Replaced Residue instance associates a specific coordinate of a protein sequence with two PSI-MOD ontology [62] attributes: the first identifies the amino acid found at that position in the normal protein and the second attribute identifies the amino acid that replaces it in the mutant protein. For example, the most frequently found mutation in EGFR is the substitution of a leucine residue at position 858 with an arginine residue in the catalytic domain of EGFR. This mutation disrupts autoinhibitory interactions, facilitating adoption of an active conformation [63]. The Reactome record for EGFR L858R (Figure 1b) indicates this amino acid substitution.

The FragmentModification subclass includes two subclasses, FragmentInsertionModification and FragmentDeletionModification. FragmentInsertionModification is used to annotate insertions of amino acid residues in a protein sequence. FragmentDeletionModification is used to annotate removal of amino acid residues. PIK3R1 Y463_L466del is a variant of the PI3K regulatory subunit p85alpha found in endometrial cancer (Figure 1c). This PIK3R1 mutant lacks four amino acid residues in the inter-SRC homology 2 (iSH2) domain. PIK3R1 is able to bind the catalytic subunit of PI3K, PIK3CA (p110alpha), but does not inhibit it, resulting in the constitutive activity of PI3K, in the absence of growth factors [64]. The deletion coordinates are indicated in the Reactome record for PIK3R1 Y463_L466del mutant.

The FragmentModification class can also be used to annotate fusion proteins. For example, the translocation t(8;22)(p11;q11) in chronic myeloid leukemia produces a BCR-FGFR1 fusion that consists of the first four exons of BCR and exons 9–18 of FGFR1 [65]. The BCR-FGFR1 fusion protein is annotated as an Entity with Accessioned Sequence (Figure 1d) that consists of a truncated BCR protein, starting at position 1 and ending at position 584 of the reference UniProt sequence P11274 (human BCR). Then, a FragmentInsertionModification instance defines insertion of amino acids 429–822 of the UniProt reference sequence P11362 (human FGFR1) at position 585 of BCR (Figure 1d).

**Figure 2:**
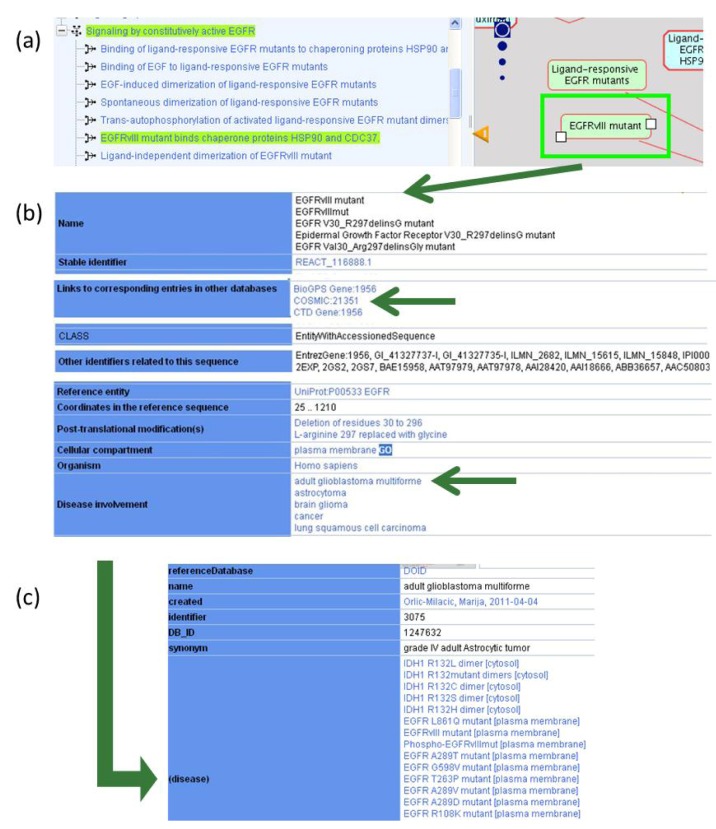
Disease information presented interactively on the Reactome website. (**a**) Selecting an entity in the pathway diagram, EGFRvIII mutant in this case and opening the Reactome details pane by clicking on the yellow triangle at the bottom of the Pathway Browser page brings up a record for the selected instance. A pathway hierarchy displayed on the left hand side shows how the selected instance is related to the rest of the pathway content. (**b**) Reactome record, displayed in the details pane, provides information on the selected entity, including cross-references to other databases such as COSMIC, UniProt, GO. (**c**) Cross-reference to Disease Ontology: clicking on a disease attribute, such as “adult glioblastoma multiforme”, provides a Disease Ontology (DO) identifier for this disease instance (3075) and lists all proteins in Reactome database associated with adult glioblastoma multiforme.

On the Reactome website, selecting a physical entity or an event node by clicking on a pathway diagram brings up a record for that particular instance in the details pane, which appears by clicking on the yellow triangle at the bottom of the Pathway Browser page. Selecting EGFRvIII in the diagram (Figure 2a), brings up Reactome information on this mutant protein, as well as interactive cross references that direct users to other Reactome website pages or other databases of interest (Figure 2b). Each cancer-related disease variant record cross-references available records in the Catalogue of Somatic Mutations in Cancer (COSMIC) database (Table 1) [66]. The EGFRvIII record displayed on Reactome website links to COSMIC record 21351, which provides information on nucleotide sequence changes and tumor samples in which this mutation was reported.

#### 2.1.2. Associating Disease Attributes with Physical Entities and Events

All physical entities related to disease variants, such as proteins, sets of proteins, and protein complexes are tagged with disease attributes (Table 1), using a term from the Disease Ontology (DO) [67]. This DO record provides, when possible, a link to the synonymous disease record in the National Cancer Institute Thesaurus (NCIt) [68]. The disease attribute of the physical entity is assigned to all reactions and pathways in which it participates.

Besides providing information on disease involvement of specific proteins and directing users to more detailed disease descriptions, a disease attribute annotation enables users to search Reactome database for proteins and events associated with a specific disease. For example, in Figure 2b, a DO instance “adult glioblastoma multiforme” is associated with EGFRvIII. Clicking on the “adult glioblastoma multiforme” link displayed on Reactome website (Figure 2b) provides a DO identifier for this disease instance (3075) and also lists all other proteins in Reactome database whose mutant forms are associated with adult glioblastoma multiforme (Figure 2c). Thus, Reactome provides cancer researchers with a quick access to cancer type-specific disease variants and information on the mechanism of action for each variant annotated.

#### 2.1.3. Mode of Action and Specificity of Anti-Cancer Therapeutics

**Figure 3:**
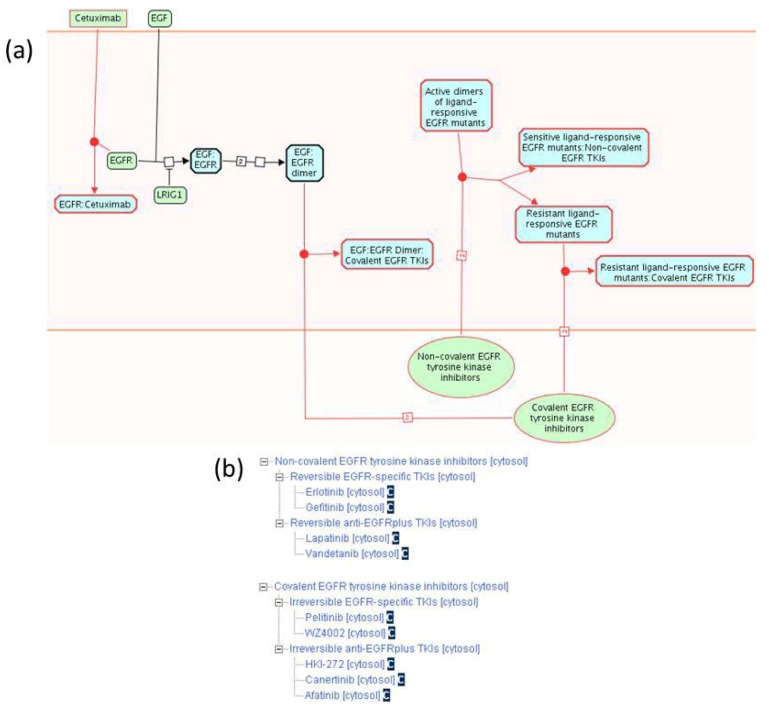
Mode of action and specificity of anti-EGFR cancer therapeutics. (**a**) Anti-EGFR therapeutics differ in their specificity for EGFR cancer variants, as well as in their mode of action (non-covalent *vs*. covalent binding). (**b**) Classification of EGFR-binding small tyrosine kinase inhibitors (TKIs) according to spectrum and reversibility of their binding.

The Reactome data model allows for annotation of small molecules and antibodies used as anti-cancer therapeutics, as well as the annotation of their specific mode of action. We have annotated nine small tyrosine kinase inhibitors (TKIs) used to inhibit EGFR kinase activity in cancer [59,69], as well as the recombinant antibody cetuximab [70] (Figure 3a). In addition, we annotated five benzaquinoid ansamycins that inhibit the HSP90 chaperone protein that stabilizes EGFR mutant proteins [71], twelve anti-FGFR TKIs [60], one anti-FGFR recombinant antibody [72], ten small molecules that inhibit the catalytic subunit of PI3K [61], and three small molecules that inhibit AKT [61] (Table 2).

For each anti-EGFR TKI, we specify whether it associates with the EGFR catalytic domain through formation of a covalent (irreversible) bond or through a non-covalent interaction (reversible). We also specify whether a TKI is EGFR-specific or whether it can inhibit other receptor tyrosine kinases besides EGFR (EGFRplus). Each small molecule instance we annotate is associated with the Chemical Entities of Biological Interest (ChEBI) database identifier [19]. On the Reactome website, a link to a corresponding ChEBI record is displayed after the name of each small molecule. Clicking on the ChEBI link associated with gefitinib (Figure 3b) directs the user to the gefitinib information in ChEBI, displaying its molecular structure and additional information not directly captured by Reactome.

EGFR cancer mutants in Reactome are classified into sets based on their sensitivity to various TKIs (Figure 3a). Ligand responsive EGFR mutants sensitive to non-covalent TKIs can be inhibited by low concentrations of non-covalent (reversible) TKIs that do not significantly affect the function of wild-type EGFR and therefore produce minimal side effects. Ligand responsive EGFR mutants resistant to non-covalent TKIs can be inhibited by covalent (irreversible) TKIs. As can be seen from the diagram (Figure 3a), concentrations of irreversible TKIs that inhibit EGFR mutants also inhibit the function of the wild-type protein, causing more severe side effects, as described in event summations. Cetuximab is used for treatment of cancers that overexpress wild-type EGFR protein, usually due to amplification of the EGFR locus [59,70].

**Table 1:** Cancer-related disease variants published by Reactome to date. A total of ~150 cancer mutants have been published since the start of the project in December 2010.

Disease Variant	COSMIC Identifier(s)	Mutation Type	Disease	Reactome Pathway Name
EGFR A289D mutant	21685	Missense	Glioblastoma	Signaling by EGFR in Cancer
EGFR A289T mutant	21686	Missense	Glioblastoma, oligodendroglioma	Signaling by EGFR in Cancer
EGFR A289V mutant	21687	Missense	Glioblastoma	Signaling by EGFR in Cancer
EGFR D770_N771insNPG mutant		Insertion	Lung cancer	Signaling by EGFR in Cancer
EGFR D770_N771insNPH mutant	48920	Insertion	Lung cancer	Signaling by EGFR in Cancer
EGFR E746_A750del mutant	6223, 129800, 6225	Deletion	Breast, head and neck, kidney, lung, ovarian, salivary gland and thyroid cancer	Signaling by EGFR in Cancer
EGFR E746_A750del; T790M mutant		Deletion; Missense	Lung cancer	Signaling by EGFR in Cancer
EGFR E746_S752delinsV mutant	18492, 18426, 12384, 85797	Deletion	Lung cancer	Signaling by EGFR in Cancer
EGFR E746_T751delinsA mutant	20845, 12678, 13549	Deletion	Head and neck, lung cancer	Signaling by EGFR in Cancer
EGFR G598V mutant	34167, 21690	Missense	Glioblastoma	Signaling by EGFR in Cancer
EGFR G719A mutant	6239, 13448	Missense	Lung cancer	Signaling by EGFR in Cancer
EGFR G719C mutant	6253, 20881	Missense	Lung cancer	Signaling by EGFR in Cancer
EGFR G719S mutant	6252, 13983	Missense	Colorectal, lung cancer	Signaling by EGFR in Cancer
EGFR L747_A750delinsP mutant	13562, 12382, 12422	Deletion	Head and neck, lung cancer	Signaling by EGFR in Cancer
EGFR L747_P753delinsS mutant	13564, 12370	Deletion	Head and neck, lung, prostate cancer	Signaling by EGFR in Cancer
EGFR L747_S752del mutant	13984, 6255	Deletion	Lung cancer	Signaling by EGFR in Cancer
EGFR L747_T751del mutant	24432, 12369, 6254, 23571	Deletion	Lung cancer	Signaling by EGFR in Cancer
EGFR L747_T751delinsP mutant	24573, 12383, 22944	Deletion	Lung cancer	Signaling by EGFR in Cancer
EGFR L858R mutant	6224, 12979	Missense	Breast, lung, ovarian, stomach, thymus and thyroid cancer, mesothelioma	Signaling by EGFR in Cancer
EGFR L858R;T790M mutant		Missense; Missense	Lung cancer	Signaling by EGFR in Cancer
EGFR L861Q mutant	6213, 13173	Missense	Lung cancer, glioblastoma	Signaling by EGFR in Cancer
EGFR M766_A767insASV mutant		Insertion	Lung cancer	Signaling by EGFR in Cancer
EGFR R108K mutant	21683, 34166	Missense	Glioblastoma	Signaling by EGFR in Cancer
EGFR T263P mutant	21684	Missense	Glioblastoma	Signaling by EGFR in Cancer
EGFR V738_K739insKIPVAI mutant		Insertion	Lung cancer	Signaling by EGFR in Cancer
EGFRvIII mutant	21351	Deletion	Lung cancer, glioblastoma	Signaling by EGFR in Cancer
BCR-FGFR1 fusion mutant		Translocation	Myeloid leukemia	Signaling by FGFR in Disease
CNTRL-FGFR1 fusion mutant		Translocation	Myeloid leukemia	Signaling by FGFR in Disease
CPSF6-FGFR1 fusion mutant		Translocation	Myeloid leukemia	Signaling by FGFR in Disease
CUX1-FGFR1 fusion mutant		Translocation	Myeloid leukemia	Signaling by FGFR in Disease
FGFR1 K656E mutant	35673	Missense	Glioblastoma	Signaling by FGFR in Disease
FGFR1 N546K mutant	19176	Missense	Glioblastoma, hypochondroplasia	Signaling by FGFR in Disease
FGFR1 P252S mutant		Missense	Melanoma	Signaling by FGFR in Disease
FGFR1 P252T mutant	12834	Missense	Lung cancer	Signaling by FGFR in Disease
FGFR1 R576W mutant	19177	Missense	Glioblastoma	Signaling by FGFR in Disease
FGFR1OP-FGFR1 fusion mutant		Translocation	Myeloid leukemia	Signaling by FGFR in Disease
FGFR1OP2-FGFR1 fusion mutant		Translocation	Myeloid leukemia	Signaling by FGFR in Disease
FGFR1c P252R mutant		Missense	Pfeiffer syndrome	Signaling by FGFR in Disease
FGFR2 K660E mutant	36909	Missense	Endometrial cancer	Signaling by FGFR in Disease
FGFR2 K660M mutant	49175	Missense	Cervical cancer	Signaling by FGFR in Disease
FGFR2 K660N mutant	49173	Missense	Endometrial cancer, Crouzon syndrome	Signaling by FGFR in Disease
FGFR2 L764fs*4STOP mutant		Frameshift	Endometrial cancer	Signaling by FGFR in Disease
FGFR2 N549H mutant		Missense	Crouzon syndrome	Signaling by FGFR in Disease
FGFR2 N549K mutant	36912, 36902	Missense	Endometrial cancer	Signaling by FGFR in Disease
FGFR2 S267P mutant		Missense	Stomach cancer, Crouzon syndrome	Signaling by FGFR in Disease
FGFR2 W290C mutant	41286	Missense	Lung cancer, Pfeiffer syndrome	Signaling by FGFR in Disease
FGFR2b P253R mutant	49170	Missense	Endometrial cancer, acrocephalosyndactylia	Signaling by FGFR in Disease
FGFR2b S252W mutant	36903, 41289	Missense	Endometrial, ovarian cancer, acrocephalosyndactylia, craniosynostosis	Signaling by FGFR in Disease
FGFR2b S373C mutant	36905	Missense	Endometrial cancer	Signaling by FGFR in Disease
FGFR2b Y376C mutant	36904, 41290	Missense	Endometrial, ovarian cancer	Signaling by FGFR in Disease
FGFR2c A314D mutant	49171	Missense	Endometrial cancer	Signaling by FGFR in Disease
FGFR2c A314S mutant		Missense	Bone development disease	Signaling by FGFR in Disease
FGFR2c A315S mutant		Missense	Syndactyly	Signaling by FGFR in Disease
FGFR2c A315T mutant	30777	Missense	Endometrial cancer	Signaling by FGFR in Disease
FGFR2c P253R mutant	49170	Missense	Endometrial cancer, acrocephalosyndactylia	Signaling by FGFR in Disease
FGFR2c S252W mutant	41289, 36903	Missense	Endometrial, ovarian cancer, acrocephalosyndactylia, craniosynostosis	Signaling by FGFR in Disease
FGFR2c S372C mutant		Missense	Beare-Stevenson cutis gyrata syndrome	Signaling by FGFR in Disease
FGFR2c W290G mutant		Missense	Crouzon syndrome, Pfeiffer syndrome	Signaling by FGFR in Disease
FGFR2c Y375C mutant		Missense	Beare-Stevenson cutis gyrata syndrome	Signaling by FGFR in Disease
FGFR3 795fs*139STOP mutant		Frameshift	Multiple myeloma, thanatophoric dysplasia	Signaling by FGFR in Disease
FGFR3 A391E mutant	721	Missense	Bladder cancer, Crouzon syndrome	Signaling by FGFR in Disease
FGFR3 G370C mutant	716, 35897	Missense	Bladder cancer, thanatophoric dysplasia	Signaling by FGFR in Disease
FGFR3 G380R mutant	24842, 24812	Missense	Bladder cancer, multiple myeloma, achondroplasia	Signaling by FGFR in Disease
FGFR3 G382D mutant	727	Missense	Multiple myeloma	Signaling by FGFR in Disease
FGFR3 K650E mutant	719, 35899	Missense	Bladder, testicular cancer, multiple myeloma, thanatophoric dysplasia	Signaling by FGFR in Disease
FGFR3 K650M mutant	720, 85791	Missense	Bladder, testicular cancer, multiple myeloma, thanatophoric dysplasia	Signaling by FGFR in Disease
FGFR3 K650N mutant		Missense	Bladder, testicular cancer, hypochondroplasia	Signaling by FGFR in Disease
FGFR3 K650Q mutant	726	Missense	Bladder cancer, hypochondroplasia	Signaling by FGFR in Disease
FGFR3 K650T mutant	731	Missense	Bladder, testicular cancer, hypochondroplasia	Signaling by FGFR in Disease
FGFR3 R248C mutant	714, 35896	Missense	Bladder cancer, multiple myeloma, thanatophoric dysplasia	Signaling by FGFR in Disease
FGFR3 S371C mutant	17461, 35898	Missense	Bladder cancer, thanatophoric dysplasia	Signaling by FGFR in Disease
FGFR3 Y373C mutant	718, 29428	Missense	Bladder cancer, multiple myeloma, thanatophoric dysplasia	Signaling by FGFR in Disease
FGFR3b G697C mutant	24802	Missense	Head and neck cancer	Signaling by FGFR in Disease
FGFR3b S249C mutant	715, 29427	Missense	Bladder, cervical, head and neck, prostate cancer, thanatophoric dysplasia	Signaling by FGFR in Disease
FGFR3c P250R mutant		Missense	Acrocephalosyndactylia, craniosynostosis	Signaling by FGFR in Disease
FGFR4 N535D mutant		Missense	Rhabdomyosarcoma	Signaling by FGFR in Disease
FGFR4 N535K mutant		Missense	Rhabdomyosarcoma	Signaling by FGFR in Disease
FGFR4 V550E mutant		Missense	Rhabdomyosarcoma	Signaling by FGFR in Disease
FGFR4 V550L mutant		Missense	Rhabdomyosarcoma	Signaling by FGFR in Disease
FGFR4 Y367C mutant		Missense	Breast cancer	Signaling by FGFR in Disease
LRRFIP1-FGFR1 fusion mutant		Translocation	Myeloid leukemia	Signaling by FGFR in Disease
MYO18A-FGFR1 fusion mutant		Translocation	Myeloid leukemia	Signaling by FGFR in Disease
TRIM24-FGFR1 fusion mutant		Translocation	Myeloid leukemia	Signaling by FGFR in Disease
ZMYM2-FGFR1 fusion mutant		Translocation	Myeloid leukemia	Signaling by FGFR in Disease
IDH1 R132C mutant	28747, 41294	Missense	Glioblastoma	The citric acid (TCA) cycle and respiratory electron transport
IDH1 R132H mutant	28746, 41291	Missense	Glioblastoma	The citric acid (TCA) cycle and respiratory electron transport
IDH1 R132L mutant	28750	Missense	Glioblastoma	The citric acid (TCA) cycle and respiratory electron transport
IDH1 R132S mutant	28748	Missense	Glioblastoma	The citric acid (TCA) cycle and respiratory electron transport
PIK3CA E542K mutant	760, 29329	Missense	Bladder, breast, cervical, colorectal, endometrial, esophageal, gallbladder, head and neck, kidney, liver, lung, ovarian, penis, pharynx, pituitary, skin sweat gland, stomach, thyroid cancer, glioblastoma, lymphocytic leukemia	PI3K/AKT Signaling in Cancer
PIK3CA E542Q mutant	17442	Missense	Breast, colorectal, endometrial, lung cancer	PI3K/AKT Signaling in Cancer
PIK3CA E542V mutant	762	Missense	Breast, colorectal, endometrial, ovarian cancer	PI3K/AKT Signaling in Cancer
PIK3CA E545A mutant	12458	Missense	Breast, colorectal, endometrial, esophageal, lung, ovarian, prostate, thyroid cancer, glioblastoma, hepatoblastoma, synovial sarcoma	PI3K/AKT Signaling in Cancer
PIK3CA E545G mutant	764	Missense	Bladder, breast, colorectal, endometrial, head and neck, larynx, pituitary, stomach cancer, myeloid leukemia, non-Hodgkin lymphoma, retinoblastoma	PI3K/AKT Signaling in Cancer
PIK3CA E545K mutant	763, 29328	Missense	Bladder, breast. cervical, colorectal, endometrial, esophageal, gallbladder, head and neck, kidney, lung, ovarian, pancreatic, penis, pharynx, skin, stomach, sweat gland, thyroid cancer, melanoma, glioblastoma, medulloblastoma, myeloma, pituitary adenoma	PI3K/AKT Signaling in Cancer
PIK3CA E545Q mutant	27133	Missense	Bladder, breast, colorectal, esophageal, head and neck, ovarian, thyroid cancer	PI3K/AKT Signaling in Cancer
PIK3CA E545V mutant	144201	Missense	Ovarian cancer	PI3K/AKT Signaling in Cancer
PIK3CA H1047L mutant	776, 30744	Missense	Bladder, breast, colorectal, endometrial, head and neck, liver, lung, ovarian, pharynx, thyroid cancer, glioblastoma, non-Hodgkin lymphoma, pituitary adenoma	PI3K/AKT Signaling in Cancer
PIK3CA H1047R mutant	775, 29325	Missense	Bladder, breast, cervical, colorectal, endometrial, gallbladder, head and neck, liver, lung, ovarian, pancreatic, pharynx, prostate, stomach, thyroid cancer, glioblastoma, medulloblastoma, melanoma, meningioma, neuroectodermal tumor, non-Hodgkin lymphoma, pituitary adenoma	PI3K/AKT Signaling in Cancer
PIK3CA H1047Y mutant	774, 29326	Missense	Breast, colorectal, endometrial, lung, ovarian cancer, glioblastoma	PI3K/AKT Signaling in Cancer
PIK3CA M1043I mutant	773, 29313, 94984	Missense	Bladder, breast, cervical, colorectal, endometrial, lung, ovarian, thyroid cancer, glioblastoma	PI3K/AKT Signaling in Cancer
PIK3CA M1043T mutant	12463	Missense	Ovarian, stomach cancer, glioblastoma	PI3K/AKT Signaling in Cancer
PIK3CA M1043V mutant	12591, 30743	Missense	Breast, colorectal, endometrial, head and neck, lung, ovarian, pharynx cancer, glioblastoma	PI3K/AKT Signaling in Cancer
PIK3CA Q546E mutant	6147	Missense	Breast, cervical, colorectal, endometrial cancer	PI3K/AKT Signaling in Cancer
PIK3CA Q546H mutant	24712, 30740	Missense	Cervical, colorectal, endometrial cancer	PI3K/AKT Signaling in Cancer
PIK3CA Q546K mutant	766, 30738	Missense	Breast, colorectal, endometrial, lung, ovarian, stomach cancer, lymphocytic leukemia	PI3K/AKT Signaling in Cancer
PIK3CA Q546L mutant	25041, 85754	Missense	Breast, colorectal, gallbladder, head and neck cancer	PI3K/AKT Signaling in Cancer
PIK3CA Q546P mutant	767	Missense	Breast, colorectal, endometrial, ovarian cancer, glioma	PI3K/AKT Signaling in Cancer
PIK3CA Q546R mutant	12459, 30739	Missense	Breast. colorectal, endometrial, prostate, stomach cancer	PI3K/AKT Signaling in Cancer
PIK3CA R38C mutant	744	Missense	Colorectal cancer	PI3K/AKT Signaling in Cancer
PIK3CA R38G mutant	40945	Missense	Glioblastoma	PI3K/AKT Signaling in Cancer
PIK3CA R38H mutant	745, 49022	Missense	Breast, colorectal, endometrial cancer	PI3K/AKT Signaling in Cancer
PIK3CA R38S mutant	87310	Missense	Stomach cancer	PI3K/AKT Signaling in Cancer
PIK3R1 D560H mutant	125378	Missense	Pharynx cancer	PI3K/AKT Signaling in Cancer
PIK3R1 D560Y mutant	335765	Missense	Glioblastoma	PI3K/AKT Signaling in Cancer
PIK3R1 G376R mutant	35827, 132923	Missense	Endometrial cancer, glioblastoma	PI3K/AKT Signaling in Cancer
PIK3R1 H450_E451del mutant	39296	Deletion	Endometrial cancer, glioblastoma	PI3K/AKT Signaling in Cancer
PIK3R1 K459del mutant	87216	Deletion	Endometrial cancer	PI3K/AKT Signaling in Cancer
PIK3R1 N564D mutant	42912	Missense	Colorectal, endometrial cancer, glioblastoma	PI3K/AKT Signaling in Cancer
PIK3R1 N564K mutant	35808	Missense	Glioblastoma	PI3K/AKT Signaling in Cancer
PIK3R1 R574_T576del mutant	87219	Deletion	Endometrial cancer	PI3K/AKT Signaling in Cancer
PIK3R1 R574I mutant	85927	Missense	Colorectal cancer	PI3K/AKT Signaling in Cancer
PIK3R1 R574T mutant	87544	Missense	Bladder, breast cancer	PI3K/AKT Signaling in Cancer
PIK3R1 Y463_L466del mutant	87228	Deletion	Endometrial cancer	PI3K/AKT Signaling in Cancer
AKT1 E17K mutant	33765, 34142	Missense	Breast, colorectal, ovarian cancer	PI3K/AKT Signaling in Cancer
PTEN R130G mutant	5219	Missense	Endometrial, lung, ovarian cancer, glioblastoma	PI3K/AKT Signaling in Cancer
PTEN R130Q mutant	5033	Missense	Breast, colorectal, endometrial, ovarian, thyroid cancer, glioma, histiocytoma	PI3K/AKT Signaling in Cancer
PTEN R130L mutant	5216	Missense	Breast, endometrial cancer, Cowden syndrome	PI3K/AKT Signaling in Cancer
PTEN C124S mutant	5224, 5271	Missense	Endometrial, thyroid cancer, glioblastoma	PI3K/AKT Signaling in Cancer
PTEN C124R mutant		Missense	Thyroid adenoma, Cowden syndrome	PI3K/AKT Signaling in Cancer
PTEN R173H mutant	5039	Missense	Endometrial, ovarian cancer, glioma	PI3K/AKT Signaling in Cancer
PTEN R173C mutant	5089, 24682	Missense	Endometrial cancer, glioblastoma, lymphocytic leukemia, melanoma	PI3K/AKT Signaling in Cancer
PTEN R173P mutant	12735	Missense	Testicular cancer	PI3K/AKT Signaling in Cancer
PTEN S170N mutant	5045	Missense	Endometrial cancer, glioblastoma	PI3K/AKT Signaling in Cancer
PTEN S170R mutant		Missense	Bannayan-Riley-Ruvalcaba syndrome	PI3K/AKT Signaling in Cancer
PTEN H123Y mutant	5078	Missense	Endometrial cancer	PI3K/AKT Signaling in Cancer
PTEN G129E mutant	28917	Missense	Endometrial cancer	PI3K/AKT Signaling in Cancer
PTEN G129R mutant	5092	Missense	Thyroid cancer, glioblastoma	PI3K/AKT Signaling in Cancer
PTEN H93Y mutant	5043	Missense	Endometrial cancer, glioma, medulloblastoma	PI3K/AKT Signaling in Cancer
PTEN H93A mutant		Missense	Cancer	PI3K/AKT Signaling in Cancer
PTEN H93R mutant	5060	Missense	Glioblastoma, autism spectrum disorders	PI3K/AKT Signaling in Cancer
PTEN H93D mutant	5283	Missense	Endometrial cancer	PI3K/AKT Signaling in Cancer
PTEN H93Q mutant	5186	Missense	Glioblastoma	PI3K/AKT Signaling in Cancer
PTEN R130P mutant	5277	Missense	Breast, endometrial, glioblastoma	PI3K/AKT Signaling in Cancer
PTEN C124F mutant	13578	Missense	Lung cancer	PI3K/AKT Signaling in Cancer
PTEN C124Y mutant	5140	Missense	Stomach cancer	PI3K/AKT Signaling in Cancer
PTEN S170I mutant	5218	Missense	Glioblastoma	PI3K/AKT Signaling in Cancer
PTEN S170G mutant	5063	Missense	Glioblastoma	PI3K/AKT Signaling in Cancer
PTEN G129V mutant	5276	Missense	Endometrial cancer	PI3K/AKT Signaling in Cancer
PTEN R130* mutant	21342, 5152	Nonsense	Cervical, colorectal, endometrial, lung, ovarian, prostate, thyroid cancer, glioblastoma, medulloblastoma, leimyosarcoma	PI3K/AKT Signaling in Cancer
PTEN R233* mutant	5154, 21343	Nonsense	Cervical, colorectal, endometrial, lung, ovarian cancer, glioblastoma, histiocytoma, lymphocytic leukemia,	PI3K/AKT Signaling in Cancer
PTEN R335* mutant	5775, 5151	Nonsense	Head and neck, stomach cancer, glioblastoma, melanoma, Burkitt lymphoma, lymphocytic leukemia	PI3K/AKT Signaling in Cancer

**Table 2:** Anti-cancer therapeutics published by Reactome to date. A total of 39 small molecule inhibitors and 2 recombinant antibodies have been published since the start of the project in December 2010.

Anti-Cancer Therapeutic	Reference Molecule Identifier	Specificity	Reactome Pathway Name
17-AAG	ChEBI^a^: 64153	HSP90	Signaling by EGFR in Cancer
17-DMAG	ChEBI:65324	HSP90	Signaling by EGFR in Cancer
Afatinib	ChEBI:61390	EGFR, ERBB2	Signaling by EGFR in Cancer
Canertinib	ChEBI:61399	Pan-ERBB	Signaling by EGFR in Cancer
Cetuximab	Recombinant antibody	EGFR	Signaling by EGFR in Cancer
Erlotinib	ChEBI:114785	EGFR	Signaling by EGFR in Cancer
Gefitinib	ChEBI:49668	EGFR	Signaling by EGFR in Cancer
Geldanamycin	ChEBI:5292	HSP90	Signaling by EGFR in Cancer
HKI-272	ChEBI:61390	EGFR, ERBB2	Signaling by EGFR in Cancer
Herbimycin A	ChEBI:5674	HSP90	Signaling by EGFR in Cancer
Lapatinib	ChEBI:49603	EGFR, ERBB2	Signaling by EGFR in Cancer
Pelitinib	ChEBI:38927	EGFR	Signaling by EGFR in Cancer
Vandetanib	ChEBI:49960	EGFR, VEGFR	Signaling by EGFR in Cancer
WZ4002	ChEBI:61400	EGFR	Signaling by EGFR in Cancer
IPI-504	Pending	HSP90	Signaling by EGFR in Cancer
AZ 2171	ChEBI:556867	FGFR, PDGFR, VEGFR. KIT	Signaling by FGFR in Disease
Brivanib	ChEBI:443041	FGFR, VEGFR	Signaling by FGFR in Disease
Brivanib alaninate	ChEBI:270995	FGFR, VEGFR	Signaling by FGFR in Disease
Dovitinib	ChEBI:594834	FGFR, FLT3, VEGFR, PDGFR, KIT, CSFR	Signaling by FGFR in Disease
E3810	Pending	FGFR, VEGFR	Signaling by FGFR in Disease
E7080	ChEBI:816009	FGFR VEGFR, PDGFR	Signaling by FGFR in Disease
Masitinib	ChEBI:63450	FGFR3, PDGFR, KIT	Signaling by FGFR in Disease
GP369	Recombinant antibody	FGFR2b	Signaling by FGFR in Disease
Midostaurin	ChEBI:63452	FGFR, FLT3, PDGFR, VEGFR, KIT, PKCA	Signaling by FGFR in Disease
PD173074	ChEBI:63448	Pan-FGFR	Signaling by FGFR in Disease
AZD4547	ChEBI:63453	Pan-FGFR	Signaling by FGFR in Disease
BGJ398	ChEBI:63451	Pan-FGFR	Signaling by FGFR in Disease
SU5402	ChEBI:63449	FGFR, VEGFR	Signaling by FGFR in Disease
GSK1059615	Pending	Pan-PI3K	PI3K/AKT Signaling in Cancer
BEZ235	Pending	PI3K Class I, mTOR	PI3K/AKT Signaling in Cancer
BGT226	Pending	PI3K Class I, mTOR	PI3K/AKT Signaling in Cancer
BKM120	Pending	PI3K Class I	PI3K/AKT Signaling in Cancer
XL765	Pending	PI3K Class I, mTOR	PI3K/AKT Signaling in Cancer
XL147	Pending	PI3K Class I	PI3K/AKT Signaling in Cancer
GDC0941	ChEBI:65326	PI3K Class I	PI3K/AKT Signaling in Cancer
PX-866	ChEBI:65345	PIK3CA, PIK3CD, PIK3CG	PI3K/AKT Signaling in Cancer
LY294002	ChEBI:65329	Pan-PI3K	PI3K/AKT Signaling in Cancer
wortmannin	ChEBI:52289	Pan-PI3K	PI3K/AKT Signaling in Cancer
Perifosine	ChEBI:428891	AKT	PI3K/AKT Signaling in Cancer
MK2206	ChEBI:716367	AKT	PI3K/AKT Signaling in Cancer
Triciribine	ChEBI:65310	AKT	PI3K/AKT Signaling in Cancer

### 2.2. Other Disease Pathways in Reactome

In addition to cancer, Reactome also collects and provides information on communicable diseases. Currently featured infection-related Reactome pathways are “HIV Infection”, “Influenza Infection”, “Botulinum Neurotoxicity”, and “Latent Infection with Mycobacterium tuberculosis”. The pathway “Signaling by FGFR in Disease” contains, besides information on FGFR in cancer, the information on FGFR mutations and their functional implication in various developmental disorders, such as Pfeiffer syndrome and Crouzon syndrome. Reactome has recently published “Abnormal Metabolism in Phenylketonuria” and “Mucopolysaccharidoses” pathways, thereby introducing metabolic genetic diseases.

### 2.3. Enhancing the Reactome Pathway Browser for Display of Disease Variants

**Figure 4:**
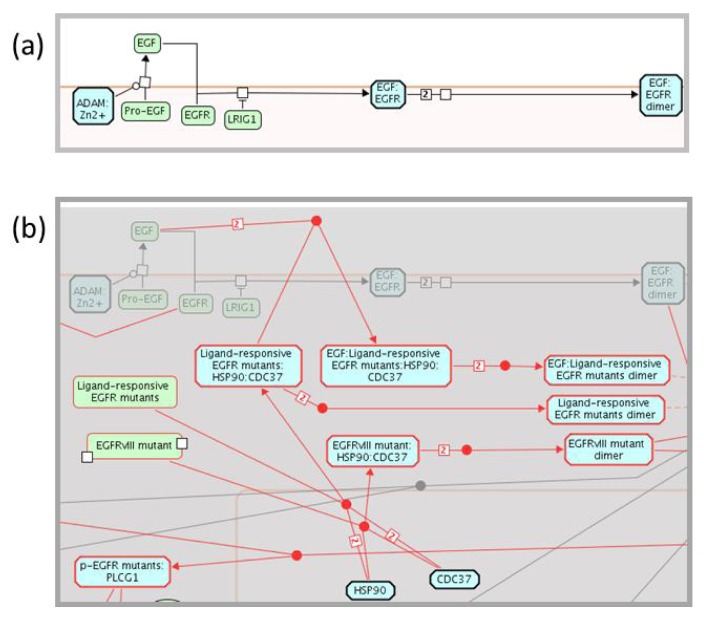
Display of wild-type and disease pathway diagrams. (**a**) A cancer disease attribute, assigned to events involved in cancer, instructs the browser to hide disease events when a user selects to view a wild-type pathway. (**b**) When a user selects to view a disease pathway, disease events appear in the wild type diagram, while all normal events are shaded. All disease events and physical entities with disease tags are outlined in red for easier visualization.

The Reactome Pathway Browser, based upon the Systems Biology Graphical Notation (SBGN) [73], permits the navigation and analysis of Reactome data, in a similar manner to Google Maps. SBGN is a standard graphical representation of biological pathway and network models. The Pathway Browser was adapted to enable display of disease variants and disease-related events involving proteins. A pathway diagram is shared between a wild-type pathway, for example “Signaling by EGFR”, and the corresponding disease pathway, “Signaling by EGFR in Cancer”. A disease attribute, attached to events involving cancer, instructs the browser to hide disease events when a user selects a wild-type pathway view (Figure 4a). When a user selects a disease pathway view, disease events appear in the diagram while all normal events are shaded gray. All disease events and physical entities with disease tags are outlined in red for easier visualization (Figure 4b).

**Figure 5:**
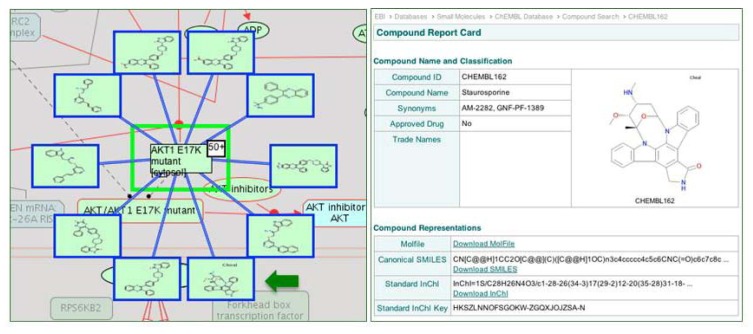
AKT1 E17K mutant-small molecule interactions. When ChEMBL is selected as the interaction database, the MI Overlay displays small molecules from ChEMBL as interactors of AKT1 E17K variant protein of the PI3K/AKT Signaling in Cancer pathway. The nodes of the mini network are interactive; clicking the node to the left of the green arrow will link out to the Staurosporine protein kinase inhibitor record at ChEMBL.

Physical entity and reaction nodes within the pathway diagrams are interactive. Clicking on either feature displays specific information and additional links out to external databases in the “Details” Panel, which opens by clicking on the yellow triangle at the bottom of the Pathway Browser page (Figure 2). Context sensitive menus, accessible through the right click on a selected entity, provide additional information about the physical entity in the pathway: a catalogue of other pathways in Reactome in which the selected entity participates; a list of the entities that contribute to the macromolecular complex; a catalogue of interactors of the selected entity; and the option to export a list of interactors of the selected entity. The latter two features of the context sensitive menu increase protein coverage and associated variant annotations. The Molecular Interaction Overlay (MI Overlay), accessible through “Analyze, Annotate & Upload” button of the Pathway Browser, displays proteins interacting with the manually annotated protein components of a Reactome pathway. This network overlay tool employs PSICQUIC (Proteomics Standard Initiative Common QUery InterfaCe) to apply an interactive display of interaction data from an external database such as IntAct [74] into Reactome pathway diagrams. Other sources of interaction data include protein-protein and protein-drug/small molecule interactions; a user-supplied list can also be displayed. By displaying interaction data from ChEMBL, a database of bioactive drug-like molecules (Figure 5) [75], the MI Overlay feature provides an opportunity to identify protein variant-drug interactions, identify novel cancer targets or off-target effects, or pharmaceuticals that can moderate perturbed reactions or pathways experimentally.

### 2.4. Reactome Cancer-Perturbed Pathways Support Pathway Visualization and Analysis

The Pathway Browser provides an intuitive and interactive pathway visualization system, promoting a variety of web-based data analyses of user-supplied experimental data. The Pathway Analysis tool provides two alternate functions to analyze lists of genes. First, in the identifier (ID) mapping mode, a user-supplied set of gene or protein identifiers can be mapped to Reactome events. Second, in the overrepresentation analysis mode, users can determine which pathways are statistically overrepresented in a gene/protein list. The Expression Analysis tool will aid with the biological interpretation of large-scale cancer genome sequencing, genomics and proteomics experiments. For example, this tool allows users to visualize expression data (or any other numeric value, e.g., differential expression) superimposed on the Reactome pathway diagram. Reactome applies an orthology-based computational algorithm to curated human data to infer pathways in 22 diverse model organisms. The Species Comparison tool allows users to visually compare and contrast human pathways with these predicted model organism pathways. As additional cancer-perturbed pathways are added to Reactome, this method of “inferred” curation will provide a platform from which to study molecular disease mechanisms across the evolutionary spectrum. Reactome data is available for downloading and manipulation by third party visualization and analysis tools, including Cytoscape, Vanted and CellDesigner [76,77,78].

## 3. Experimental Section

Using the previously curated human EGFR pathway, which included a number of annotations for EGFR and downstream signaling by SHC1, GRB2, PLCG1 and CBL, as a template from which to extend the EGFR pathway, we imported this dataset into the Reactome Curator Tool [11]. Briefly, the curator tool provides Reactome curators with all the necessary tools to access the Reactome database and annotate data in agreement with the Reactome data model. Curators identified research articles and reviews in PubMed that were relevant to the annotation of the cancer-perturbed EGFR, FGFR and PI3K/AKT pathways. Once publications had been reviewed, a list of cancer-related proteins, small molecules and macromolecular complexes was prepared. Additional queries were performed in UniProt and ChEBI to identify the reference entity proteins and small molecules, respectively that would be used to construct the reactions of the cancer-perturbed EGFR pathway. Additional attributes of a reaction were captured. For example, details of the input and output entity(s), the catalytic or regulatory protein(s), the cellular location(s) of the reactants, a textual summation describing the reaction and the supporting literature reference(s). The Disease Ontology terms that match literature references and COSMIC records for annotated cancer variants were assigned as disease attributes to physical entities and events involving these mutant proteins. Oncogenic overexpression of proteins as a consequence of gene amplification is usually not explicitly shown in pathway diagrams, but is captured in text summations that accompany cancer pathways.

## 4. Conclusions

Reactome is a highly reliable, curated database of biological pathways. Through our website, we provide access to pathway and network data analysis tools for visualizing pathway data and interpreting experimental data sets. All Reactome data and software is openly available with no licensing required.

In view of the potential applicability of pathway and network analyses to identify and characterize novel cancer targets, Reactome has integrated and expanded the pathway gene product-function annotation and pathway curation to promote comprehensive and effective characterization of cancer targets, their related relationships and pathways. Our curation efforts thus far have focused on the EGFR pathway (including the EGFR, ERBB2, ERBB3, ERBB4 receptors), FGFR and PI3K-AKT signaling and their downstream effector genes. Reactome curators will enhance our curation of other cancer-perturbed pathways, such as apoptosis, cell cycle checkpoints, and other signaling pathways, including BMP, PDGF, NOTCH, VEGF, WNT, Rho-GTPase, and TGF-beta. Furthermore, as the Ontario Institute for Cancer Research and its partners in the International Cancer Genome Consortium (ICGC) [79,80] sequence various tumor genomes, new cancer-related candidate pathways will be identified and curated into Reactome. Existing Reactome pathways are updated on a regular basis, and additional cancer variants and anti-cancer drugs implicated in EGFR, FGFR and PI3K/AKT pathways will be included as information on their function becomes available.

Reactome is not the only pathway database to curate pathway data relevant to cancer and disease. Cancer-perturbed signaling pathways can be found in KEGG, Panther, MetaCyc, and NCI-PID [81,82,83,84]. The Reactome data model, however, provides a more detailed framework for the curation of the knowledge relevant to cancer-related pathways, a visualization environment to display pathway data, and a suite of analysis tools for the interpretation of experimental cancer data sets.

A number of other bioinformatics databases such as Mouse Genome Informatics (MGI) [85] and Comparative Toxicogenomics Database (CTD) [86] have established disease curation pipelines, employing OMIM. OMIM is a detail-orientated database of disease annotation, widely used by the clinical community, but it lacks the structure and features of an ontology that would otherwise make it a perfect data source to systematically reference disease. Curation of human disease requires an establishment of a widely accessible and structured vocabulary (or ontology) that consists of knowledge that is familiar to Reactome’s end user, flexible to future Reactome annotation updates, and open to semantic reasoning. One such ontology is the Disease Ontology. Reactome will continue to work with the research community to support the development and continuous improvement of human disease ontologies and will link out to the relevant cancer and disease-related databases, to advance our own annotation consistency. In future versions of Reactome, we may also cross-reference NCIt [68] directly for cancer-related physical entities and events. The Disease Ontology does provide NCIt identifiers when possible, but disease terms captured by the Disease Ontology and NCIt do not completely overlap. Cross-referencing different ontologies will make our disease annotations more comprehensive and stable. Since some amount of overlap exists between disease terms in any disease ontology, the overlap is reflected in our current annotation of disease attributes. This is not ideal and we are developing guidelines to standardize the use of disease terms in Reactome. As far as anti-cancer therapeutics are concerned, we do not capture their approval for clinical use other than in text summations, as this is outside the scope of Reactome project. However, cross-referencing a drug database, such as PharmaGKB [87] would provide Reactome users with easy access to clinically relevant drug information, and is currently under our consideration.

We are working on further improvements to the Reactome pathway browser to produce more compact images and to be able to share one diagram between the wild-type pathway and several disease pathways with different etiologies. Furthermore, we are making additions to the Molecular Interaction Overlay to promote visual linkages between pathway entities and disease annotations, such as OMIM. Network-based methods have been used extensively in genomic and proteomic studies to analyze challenging and complex datasets. Reactome provides the Functional Interaction (FI) network plug-in for Cytoscape, which can identify network patterns related to diseases, including cancer [88]. Future expansion of the FI network with interactions based upon Reactome cancer-related pathways should significantly improve coverage, enhance the functionality of the analysis, and enrich the functional annotations supported by the FI network plug-in. Reactome will continue to develop novel and useful technologies for the querying, visualization and analysis of experimental datasets, in the context of not only normal but also disease pathways.
